# Multi-View Soft Attention-Based Model for the Classification of Lung Cancer-Associated Disabilities

**DOI:** 10.3390/diagnostics14202282

**Published:** 2024-10-14

**Authors:** Jannatul Ferdous Esha, Tahmidul Islam, Md. Appel Mahmud Pranto, Abrar Siam Borno, Nuruzzaman Faruqui, Mohammad Abu Yousuf, AKM Azad, Asmaa Soliman Al-Moisheer, Naif Alotaibi, Salem A. Alyami, Mohammad Ali Moni

**Affiliations:** 1 Department of Information and Communication Technology, Bangladesh University of Professionals, Mirpur Cantonment, Dhaka 1216, Bangladesh; jannatul.ferdous.esha11235@gmail.com (J.F.E.); itahmidul146@gmail.com (T.I.); amhpranto@gmail.com (M.A.M.P.); abrarsiam801@gmail.com (A.S.B.); 2 Department of Software Engineering, Daffodil International University, Daffodil Smart City, Birulia 1216, Bangladesh; faruqui.swe@diu.edu.bd; 3 Institute of Information Technology, Jahangirnagar University, Dhaka 1342, Bangladesh; 4 Department of Mathematics and Statistics, Faculty of Science, Imam Mohammad Ibn Saud Islamic University (IMSIU), Riyadh 13318, Saudi Arabia; kazad@imamu.edu.sa (A.A.); asmaalmoisheer@gmail.com (A.S.A.-M.); nmaalotaibi@imamu.edu.sa (N.A.); saalyami@imamu.edu.sa (S.A.A.); 5 AI & Digital Health Technology, AI and Cyber Futures Institute, Charles Sturt University, Bathurst, NSW 2795, Australia; 6 AI & Digital Health Technology, Rural Health Research Institute, Charles Sturt University, Orange, NSW 2800, Australia

**Keywords:** disability research, lung cancer, attention mechanism, convolutional neural networks, image classification

## Abstract

**Background:** The detection of lung nodules at their early stages may significantly enhance the survival rate and prevent progression to severe disability caused by advanced lung cancer, but it often requires manual and laborious efforts for radiologists, with limited success. To alleviate it, we propose a Multi-View Soft Attention-Based Convolutional Neural Network (MVSA-CNN) model for multi-class lung nodular classifications in three stages (benign, primary, and metastatic). **Methods:** Initially, patches from each nodule are extracted into three different views, each fed to our model to classify the malignancy. A dataset, namely the Lung Image Database Consortium Image Database Resource Initiative (LIDC-IDRI), is used for training and testing. The 10-fold cross-validation approach was used on the database to assess the model’s performance. **Results:** The experimental results suggest that MVSA-CNN outperforms other competing methods with 97.10% accuracy, 96.31% sensitivity, and 97.45% specificity. **Conclusions:** We hope the highly predictive performance of MVSA-CNN in lung nodule classification from lung Computed Tomography (CT) scans may facilitate more reliable diagnosis, thereby improving outcomes for individuals with disabilities who may experience disparities in healthcare access and quality.

## 1. Introduction

Lung cancer remains one of the leading causes of cancer-related deaths worldwide, with an estimated 1.8 million fatalities and 2.2 million new cases reported in 2020 alone. Despite advancements in medical imaging and diagnostic tools, early detection of lung nodules, which is crucial for improving patient survival rates, remains a significant challenge [1,2]. This difficulty arises due to the subtle nature of early-stage nodules and the labor-intensive process required for manual detection by radiologists. Non-Small Cell Lung Cancer (NSCLC), which includes subtypes such as squamous cell carcinoma, large cell carcinoma, and adenocarcinoma, accounts for approximately 80–85% of all lung cancer cases. Early and accurate classification of these nodules is essential to mitigate disease progression and improve patient outcomes [3,4].

The fundamental issue with lung cancer is that it takes a long time for symptoms to start showing up once the disease has progressed. Early identification of lung nodule cancer is critical for lowering the disease’s incidence and extending patients’ life expectancy. Cancer of the lungs can be found using seven distinct procedures: chest radiographic cytology sputum and Chest X-Rays (CXRs), Positron Emission Tomography Positron Emission Tomography (PET) scans, breath analysis, CT scans, cytology sputum, and Magnetic Resonance Imaging Magnetic Resonance Imaging (MRI) [5]. The radiography screening performance depends on the radiologists’ ability to spot abnormal lung nodules, and it becomes incredibly challenging to detect the smaller lung nodules manually. Thus, a radiologist’s or doctor’s labor and the time it takes to analyze each slice will increase. It is also conceivable that using this manual detection approach would lead to missed or incorrect diagnoses of nodule categorization and require a nodule classifier to classify the nodules based on their features.

Computer-aided diagnosis systems, an automated image categorization approach to detect lung nodules, may assist radiologists in the classification accuracy, speed up diagnosis, and increase diagnostic confidence. Standard computer-aided diagnosis systems often face challenges with accurately segmenting and classifying small nodules, which can be difficult to detect because of their size and the way they appear on CTscans. Studies have pointed out that this limitation in segmentation can lead to misclassifications, making it easy to miss tiny nodules [6,7,8]. Traditional Computer-Aided Diagnosis (CAD) technology struggles with a high rate of false positives when detecting nodules, leading to reduced accuracy in distinguishing between malignant and benign nodules. Further image processing modules, including lung nodule segmentation, CTimage modifications, and feature extraction, are required for a more complex procedure. A multi-group patch-based training system using aConvolutional Neural Network (CNN) outperforms the Computer-Aided Detection (CADe) approach [6]. Though the false-positive rate decreased, it had no substantial effect. Using CNN and Support Vector Machine (SVM) techniques, lungCTscans are classified as squamous cell carcinoma, adenocarcinoma, normal, or large cell carcinoma [7]. The approach was evaluated using chest CT-scan image sample data, a commonly used and accessible set of scans. The suggested hybridCNN-SVM model was evaluated on 5103 images, revealing its benefits and possible applications. Both CNN and SVM had an acceptable accuracy, although the test pictures were limited, and just one publicly accessible dataset was used for classification. Da et al. [8] proposed aCNN-based Computer-Aided Diagnosis (CADx) system for classifying lung nodules on CT scans, achieving a high accuracy above 91% and emphasizing the elimination of feature extraction processes to enhance efficiency. Zhang et al. [9] developed a CAD system utilizing 3D Deep Convolutional Neural Network (DCNN) for nodule identification, highlighting the need for further enhancement, especially in detecting tiny nodules. Li et al. [10] introduced a CAD system for lung nodule detection usingCTscans, focusing on enhancing static image detectors to learn relevant information while retaining details about tiny nodules.

Most models classify suspected lung nodules as malignant or benign using binary classification [11,12,13,14,15,16,17,18,19,20,21,22,23,24]. Many of these models are unable to divide nodules into stages or map malignant features. A deep learning model that can swiftly and accurately classify multi-stage nodules is needed, especially in clinical settings. Integrating clinical diagnostic data into such models improves decision-making and disease pathology insights. Soft attention mechanisms improve interpretability and transparency, helping researchers understand nodules’ complex malignancy. Custom weight settings are also important for model accuracy, especially in multi-class classification. The performance of state-of-the-art models shows the practicality and efficiency of these advancements, promising effortless incorporation into regular clinical settings. A specially created neural network model has been included for the purpose of classifying lung nodules in CT images. This model, in contrast to previous methods, combines a soft attention mechanism, batch normalization, dropout, and special convolutional layers. This arrangement concentrates on the most pertinent portions of the picture, which improves feature extraction and classification accuracy. These architectural improvements outperform conventional approaches by better addressing the problems associated with lung nodule diagnosis, such as class imbalance and limited data. The following are the primary contributions of this research work.

- A novel MVSA-CNN model was developed that applies multi-view CT scan inputs to improve lung nodule classification accuracy across different stages.
- The soft-attention mechanism was used to prioritize fundamental features in CT scans, which improved both interpretability and classification performance.
- Custom class weights were applied to decrease class imbalance, significantly improving the sensitivity and specificity in minority classes.
- Clinical diagnostic data were integrated into the model to align predictions with real-world clinical decision-making, improving diagnostic accuracy.
- The proposed model demonstrates superior performance over existing state-of-the-art models, achieving higher accuracy, sensitivity, and specificity.

The remainder of this paper is organized as follows: Section 2 reviews related works in the field of lung nodule classification and highlights their limitations. Section 3 describes the proposed methodology, including data preprocessing, feature extraction, and the architecture of the multi-view soft attention-based model. Section 4 presents the experimental results, evaluation metrics, and performance comparisons with state-of-the-art methods. Section 5 discusses the limitations of the proposed model and potential future directions.

## 2. Literature Review

Medical image analysis has shown remarkable outcomes using deep learning algorithms in recent years. For medical image analysis, a convolutional neural network is more efficient than any other architecture, as it gives a better accuracy rate and better performance than all other algorithms.

Expanding on the advancements in medical image analysis facilitated by CNN [11,12,13,14,18,19,20,21,22,23,24], researchers have explored diverse deep neural network architectures such as Long Short-Term Memory (LSTM), Recurrent Neural Network (RNN), andCNN for specific tasks like lymph node categorization [25]. While LSTM andCNN classifiers have shown superior performance over RNN models, challenges persist regarding their clinical utility due to sub-optimal efficiency. Additionally, initiatives like DeepLung [26] and attention mechanisms [27] have been introduced for lung nodule identification and classification, showcasing the utilization of innovative network structures and attention mechanisms to enhance diagnostic accuracy. Further innovation is evident in the development of GAN architectures like MED-GAN [28], aimed at generating high-quality 3D bone shapes from 2D X-ray images. Despite challenges posed by limited dataset size and noisy images, such approaches demonstrate promising potential for improving diagnostic imaging quality.

Liu et al. [16] proposed a MVSA-CNN for cancer categorization in the LIDC-IDRI dataset, achieving notable performance across binary and multi-class nodule views. However, ternary classification performance was weak. Abid et al. [17] developed a deep learning system for lung nodule classification, considering size, form, and cross-slice changes. The model exhibited strong performance on two datasets across multiple assessment parameters. The Multi-Crop Convolutional Neural Network (MC-CNN) model, incorporating detailed nodule information via the multi-crop pooling method, facilitated a non-invasive suspiciousness assessment before invasive procedures. While promising, automated nodule identification is essential to enhance diagnostic efficiency, given the increasing volume of medical radiological sequences. Tran et al. [15] introduced LdcNet, a 2D deepCNN structure for identifying lung nodule candidates inCTscans, employing automated feature extraction and classification algorithms. The model has potential improvements through further exploration of 3DCNNs and lung volume segmentation algorithms, highlighting avenues for enhancing lung nodule characterization and diagnosis accuracy.

Moreover, Faruqui et al. [29] introduced LungNet, a neural network model combining computed tomography and portable sensor-based Medical IoT (MIoT) data to classify five types of lung cancer with low false positives and high efficiency. Utilizing a 22-layerCNN architecture, LungNet achieves 91.6% accuracy in sub-classifying stage 1 and 2 lung tumors. Xie et al. [30] developed the Multi-view Knowledge-based Collaborative (MV-KBC) model, which considers nodule heterogeneity and employs adaptive weighting and semi-supervised learning to distinguish benign and malignant lung nodules without extensive data annotation. Xie et al. [23] utilized the semi-supervised adversarial classification approach for suspicious lesion classification, achieving good accuracy on the Lung Image Database Consortium and Image Database Resource Initiative (LIDC-IDRI) dataset. Wang et al. [18] proposed an Multi-view Convolutional Neural Network (MV-CNN) for lung nodule identification, capturing nodule-sensitive features in CT scans from three angles simultaneously using a network architecture comprising threeCNN branches. Rehman et al. [31] used traditional machine learning with Local Binary Pattern (LBP) and Discrete Cosine Transform (DCT) for feature extraction, achieving good accuracy but struggling with certain cancer types due to feature overlap. To improve performance, Bushara et al. [32] employed a deep learning model with data augmentation, achieving 95% accuracy, though it requires extensive data and computational resources. Raza et al. [33] introduced Lung-EffNet, a model that uses the EfficientNet architecture to classify lung cancer fromCTscans with impressive accuracy. It addresses data imbalance and is efficient enough for potential clinical use. However, it relies heavily on good-quality data and requires careful tuning, which can be challenging for real-world implementation.

Complementing these deep learning-based approaches, initiatives like that proposed by Miah et al. [34] emphasize the importance of robust preprocessing techniques in lung cancer detection. By incorporating image processing algorithms for preprocessing and segmentation followed by neural network detection, such systems aim to improve accuracy, particularly in distinguishing malignant tumors in 3D lung CTscans. Shen et al. [14] employed MC-CNN to classify lung nodules as malignant or benign, leveraging multi-crop pooling for deep feature learning. The model suggests the efficacy of MC-CNN in categorizing nodule malignancy suspicions and its potential utility in analyzing various nodule-related features, contributing to enhanced lung cancer diagnosis accuracy through a combination of deep features and conventional image characteristics. The following Table 1 summarizes the contributions and limitations of relevant studies in this area.

Previous research has made notable advancements in lung nodule classification. However, significant challenges remain, including the complexity of the networks, difficulties in accurately detecting small nodules, and the limited size of available datasets, which still impact the effectiveness of these methods.

## 3. Proposed Methodology

**Figure 1:**
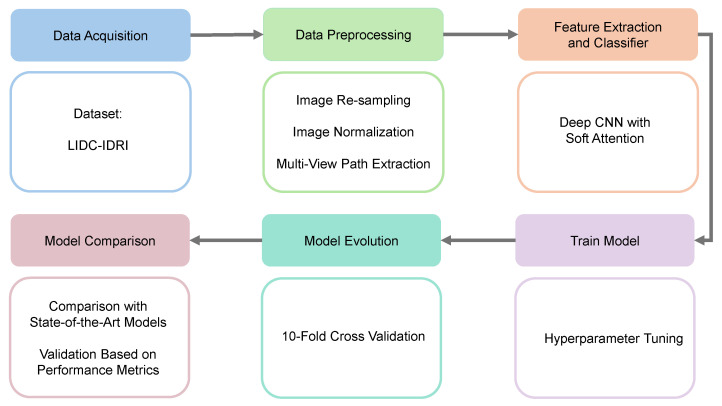
Workflow of our proposed work. (1) Data acquisition, (2) data preprocessing, (3) feature extraction and classifier, (4) train model, (5) model evolution, and (6) model comparison.

The methodology of the proposed model includes five main components: setting up the experimental framework, providing a comprehensive description of the dataset, implementing detailed data preprocessing techniques, extracting key features from the data, and performing the classification of lung nodules based on the extracted features. Figure 1 outlines the key stages of the proposed deep learning framework for lung nodule classification.

The suggested lung nodule categorization approach consists of many important steps. Initially, CT scans are collected from the LIDC-IDRI collection. These images include thorough scans annotated by several radiologists. The obtained images then go through a number of preprocessing operations to improve the quality and normalize the data, including multi-view path extraction, normalization, and resampling. Then, a deep CNN with soft attention is used to select the most relevant areas of the preprocessed pictures for feature extraction. The model is then thoroughly trained with hyperparameter adjustments made to enhance performance. After that, the model is assessed using 10-fold cross-validation, which ensures robust generalization. A performance measure-based comparison with state-of-the-art models is conducted to validate the effectiveness of the proposed approach.

### 3.1. Dataset

In this study, we used the LIDC-IDRI dataset [36,37], which is a collaboration of eight medical imaging institutions. Seven academic centers produced the LIDC-IDRI dataset, which includes 1018 cases and emerges with an Extensible Markup Language (XML) file that contains the outcome of a two-phase image annotation procedure carried out by four thoracic radiologists. A team of four experienced thoracic radiologists collaborated on these annotations in two stages. Each radiologist individually categorized each nodule into three groups in the first stage (nodule equal or larger than 3 mm, nodule less than or equivalent to 3 mm, non-nodule equal to or greater than 3 mm). Next, each radiologist assessed their annotations and those of the others anonymously. Consequently, all four radiologists analyzed each nodule annotation separately. The collection contains 244,527 images, including 1018 CT scans from 1010 people. Diagnoses can be made using this dataset. Clinical diagnosis data for the LIDC-IDRI dataset are available for 157 patients. At the patient and nodule levels, diagnostic information was gathered, where the lesions in the lungs were labeled at each level given below:

0—Unknown
1—Benign or non-malignant disease
2—Malignant, primary lung cancer
3—Malignant metastatic

### 3.2. Data Preprocessing

Data preprocessing improves the image data by removing unwanted distortions or enhancing qualities so that effective information is used for model training. The details of each preprecessing step are outlined below:

#### 3.2.1. Resampling and Normalization

The CT slices of the LIDC-IDRI dataset have variable voxel spacing. Applying spline interpolation, these CT scans were resampled to a consistent voxel spacing of 1 mm × 1 mm × 1 mm [14]. The LIDC-IDRI dataset CT slices are in the Digital Imaging and Communications in Medicine (DICOM) format, with a pixel size of 512 × 512. The slices were first converted to Hounsfield Unit (HU), which is a measurement of radiodensity. The rescale slope (0028, 1052) and rescale intercept (0028, 1053) are two pieces of information that are embedded in the DICOM file header. CT slices are converted to HU using Equation (1) below. HU of different substances are shown in Table 2 [38].

(1) HU=PixelValue×RescaleSlope+RescaleIntercept

**Figure 2:**
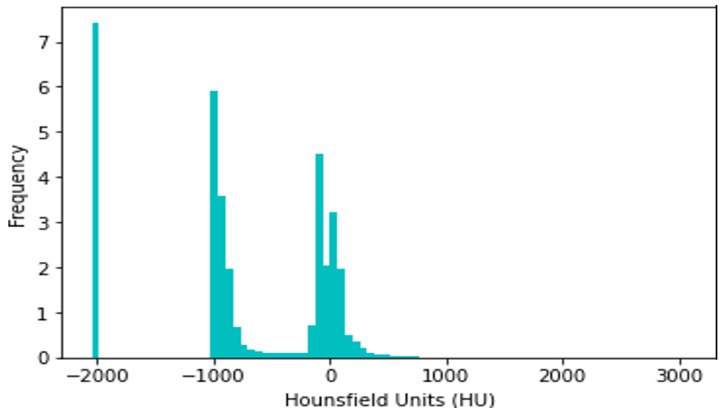
Histogram of radiodensity of LIDC-IDRI-1011.

CT slices were normalized between −1000 and +400 HU, since HU > 400 simply corresponds to bone with different radiodensity. For example, The radiodensity values in a CT slice from the LIDC-IDRI dataset are distributed according to HU, as shown in Figure 2. The differing densities of the various tissues in the scan are displayed in this histogram, where denser structures such as bone are seen at higher HU values, soft tissues are close to 0 HU, and air is about −1000 HU. Understanding this distribution is critical for the normalization procedure, since it assures that all CT slices have identical radiodensity ranges (−1000 to +400 HU). This method of data normalization helps to eliminate variances caused by different scanning circumstances and equipment, improving the accuracy and robustness of the lung nodule classification model.

#### 3.2.2. Multi-View Patch Extraction

At first, the nodule centroid coordinates (x, y, z) were determined from the corresponding XML document. Then, a 64 × 64 × 64 cube encapsulating the whole nodule was extracted for each of them. Then, from each VOI, a 2D slice of 64 × 64 size of axial, sagittal, and coronal views was extracted. Each 2D slice was resized to 128 × 128 pixels using the pillow library [39]. Therefore, a total of 3192 slices were extracted from the 157 cases for benign, primary, and metastatic lung nodules.

#### 3.2.3. Image Data Augmentation

**Figure 3:**
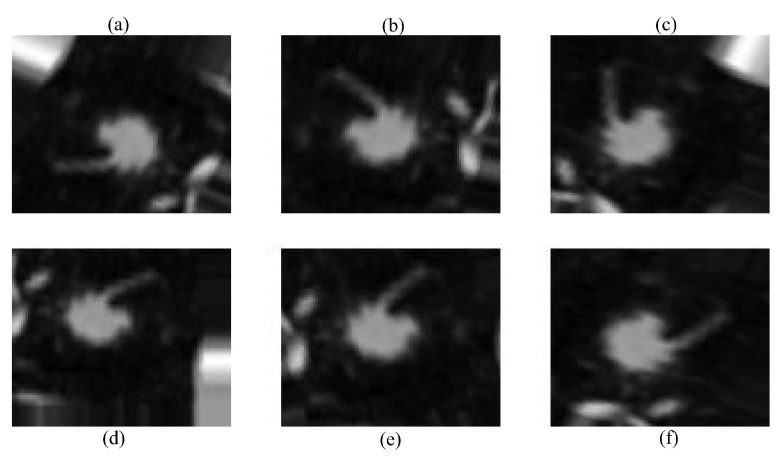
Augmented images using different techniques: (**a**) original image, (**b**) random rotation, (**c**) horizontal flip, (**d**) vertical flip, (**e**) translation, and (**f**) random zoom.

Data augmentation was used to compensate for the short dataset size and class imbalance, which might otherwise lead to overfitting. Random rotation, translation, horizontal/vertical flipping, and zooming were used dynamically during training. The robustness and generality of the model were improved using these techniques, which included diversity in the direction, location, and scale of lung nodules. This data augmentation step is illustrated in Figure 3.

Several data augmentation techniques were applied to improve the model’s robustness. First, random rotation was used to slightly rotate the CT images, ensuring recognition of lung nodules regardless of their orientation in the scan. Second, translation was applied by shifting the images in different directions, helping the model handle the natural variability in nodule placement. After that, mirrored copies of the scans were produced via horizontal and vertical flipping, which increased the amount of data and improved the generalization, respectively. Lastly, given the variety of nodule presentations in the dataset, random zooming was applied to modify the nodule sizes. This is necessary for identifying nodules of different sizes. The methods chosen were meant to resemble variances in medical imaging that occur in real life. Throughout the training process, augmentation was performed dynamically to expose the subject to a variety of settings, preventing overfitting and improving performance overall.

#### 3.2.4. Data Splitting

In this study, we used all the patient data of the LIDC-IDRI dataset, where clinical diagnosis data were available, containing 135 nodules. Next, an initial set of 3192 nodule slices was generated before augmentation. These image patches were partitioned into training and testing datasets with an 80:20 ratio, where the training data were further split into an 80:20 ratio for training and validation to aid the hyperparameter tuning process during model training. After successfully splitting the data, there were 1914 training sessions, 639 validation sessions, and 639 testing nodule patches.

### 3.3. Proposed Architecture

**Figure 4:**
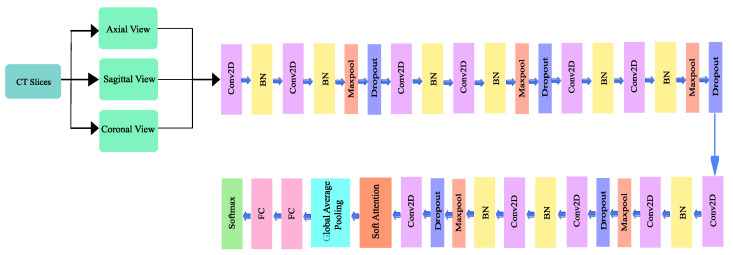
Architecture of the proposed model.

A deep convolutional neural network architecture MVSA-CNN that classifies lung nodules into “Benign”, “Primary Malignant”, and “Metastatic Malignant” phases was enhanced with a unique multi-view soft attention mechanism. With its 35 layers, each layer learns hidden characteristics that increase the nodules’ malignancy. The network may prioritize characteristics that indicate malignancy while suppressing those that produce noise during classification thanks to the soft attention mechanism, which dynamically modifies the weights allocated to various regions of the CT images. To further address class imbalances, bespoke class weights were added, and sophisticated data augmentation techniques were used to improve the model’s resilience and generalization abilities. With these changes, the model performs far better than with conventional CNN techniques. The architecture of the proposed method is illustrated in Figure 4.

#### 3.3.1. Network Design

The proposed architecture has 10 convolutional layers, including a 128 × 128 × 1 input layer. A batch normalization layer followed each odd-numbered convolution layer, and a mixture of a batch normalization layer, a max pooling layer, and a dropout layer followed each even-numbered convolutional layer. Since a higher number of layers can introduce an overwhelming amount of trainable parameters, thereby causing probable overfitting, a limited number of convolutional layers was introduced in this study. The output of the final layer neurons can be expressed using Equation (2):

(2) $$y_{i}=f\left(\sum_{i}x_{i}\times w_{i}+b_{i}\right)$$

where $y_{i}$ is the output feature map of the *i^th^* input, the activation function softmax is represented as $fx_{i}$, $w_{i}$, $b_{i}$, which are the input feature maps, weights, and bias factors, respectively. The quantity of trainable parameters for the convolutional network can be reflected using Equation (3):

(3) $$P_{i}=\left(m\times n\times l+1\right)\times k$$

where $P_{c}$ represents the number of parameters in the convolutional layer, m×n represents the kernel size, *l* is the stride, and *k* is the number of channels.

After each convolutional layer, Rectified Linear Unit (ReLU) was used as an activation function, which allows for easier and faster optimization [40]. The ReLU activation function conducts a threshold operation on each input element, setting all values below zero to zero. The operation of the ReLU can be expressed using Equation (4).

(4) $$y\left(x\right)=max\left(0,x\right)=x_{i}\;if\;x\geq 0$$

**Figure 5:**
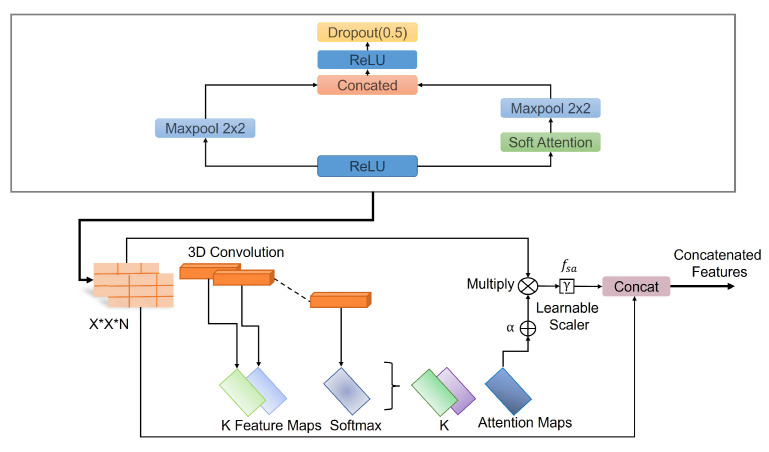
Schematic of the soft-attention block, featuring 3D convolution, softmax, learnable scaler, and concatenation operations.

The batch normalization layer was used to introduce some regularization and speed up the training process for each mini batch. The value of each mini batch is 64. This normalization aims to standardize each mini batch’s inputs before sending them to a new layer. The problem of internal covariate shift could be solved efficiently using this layer. Dropout layers with different dropout rates were used after every two convolutional layers. The main purpose of the dropout layers was to introduce regularization and minimize the possibility of over-fitting. During the weight update process, the dropout layer eliminates a random subset of parameters that prevents the model from possible over-fitting scenarios. After the 9th convolutional layer, the soft attention block was introduced. The purpose of the soft attention block was to emphasize the features that contribute to the malignancy of the nodules and suppress the elements that cause noise. A modest number of pixels determines the malignancy of lung nodules. In the soft attention block, the salient features were multiplied with higher weights, enabling more focused information. The noise-inducing features in the low-attention areas were multiplied by weights close to zero to pay less attention. The architecture of the soft attention block is illustrated in Figure 5.

The output of the soft attention block can be expressed using Equation (5).

(5) $$f_{sa}=\gamma t\left(\sum_{k=1}^{K}softmax\left(W_{k}\times t\right)\right)$$

The feature tensor *t* with weight $W_{k}$ is fed to a convolutional 3D layer. Using the *softmax* function, the output of the layer is normalized to generate k=20 attention maps. All of the attention maps were merged to create a single attention map that serves as a weighting function α multiplied with *t* to provide higher weights to the salient features that contribute to the classification of that nodule. This value is further multiplied by the scalar γ. The original features were concatenated with the retrieved feature values to generate a residual branch. During training, the value of γ was set to 0.003 so it could learn how much attention was to be incorporated in the network. The soft attention block was incorporated when the feature size was 8 × 8. After flattening the features, a dense layer containing 128 neurons and an output layer containing four neurons were introduced, followed by the softmax function. Then, a 128-neuron layer and a 4-neuron output layer were added, followed by the softmax function, which can be expressed using the following Equation (6).

(6) $$y=\frac{e^{zi}}{\sum_{j=1}^{k}e^{zj}}$$

Here, *K* represents the number of classes, and $e^{z_{i}}$ is defined as the standard exponential function for the output vector. In most cases, layer weights are initialized using the ‘Xavier Algorithm’. In the proposed work, we used a ‘He Uniform’ weight initializer that enabled increased validation accuracy and less validation loss. The unique strength of this model lies in its ability to analyzeCT scan images from multiple angles—axial, coronal, and sagittal. This multi-view approach helps the model capture subtle differences in nodule characteristics that might be missed if only a single view was used. Additionally, the soft attention mechanism allows the network to focus on the most relevant parts of the images, making it both more accurate and easier to understand. This design choice was made to address common issues in medical imaging, like data imbalance and small dataset sizes, and ultimately leads to better performance compared to traditional methods.

#### 3.3.2. Training Process

The proposed model is trained and tested on the LIDC-IDRI dataset. The model was trained on patients where clinical diagnosis data were available. The total number of nodule patches was split into an 80:20 train–test ratio, where the training data were further divided into an 80:20 training–validation ratio. The Adam optimizer was considered with an initial learning rate $=3\times 10^{-4}$ and epsilon $=1\times 10^{-8}$. There are three classes in this proposed model: benign, primary, and metastatic, and the weights for them were set as 2.5, 1, and 1.14, respectively, after extensive empirical evaluation. Categorical cross-entropy was employed as the loss function in this experiment. The working of the categorical cross entropy can be expressed using Equation (7).

(7) $${\mathrm{loss}}_{\mathrm{CCE}}=-\sum_{j=1}^{C}t_{i}log\left(f\left(s_{i}\right)\right)$$

The size of the mini-batch was set at 64. For the training process, the initial epoch number was set at 150. Early stopping was set with a patience of 25 epochs and a minimum delta of 0.001. During the 10-fold cross-validation, the improvement in accuracy became smooth almost after 40 epochs, as shown in the graph, and the training process became saturated after approximately 120 epochs. This stability in accuracy successfully concluded the training process. Table 3 contains specifics on the training parameters for the suggested model, such as the beginning epochs, early stopping criteria, number of classes, class weights, optimizer settings, learning rate, epsilon, and mini batch size.

A grid search technique was used to integrate hyperparameter adjustment in order to maximize the performance of the suggested model. A variety of hyperparameters such as batch size [32, 64, 128], dropout rate [0.2, 0.3, 0.4], and learning rate [0.0001, 0.0003, 0.001, 0.01] were methodically investigated. For each combination, the model was trained using early stopping criteria, where training was discontinued if the validation loss did not improve after 25 consecutive epochs. Because of its ability to handle sparse gradients well, the Adam optimizer was chosen and refined during the grid search process. Based on the maximum validation accuracy and lowest validation loss seen during 10-fold cross-validation, the optimal hyperparameters were selected. This systematic approach ensured a comprehensive evaluation of the model’s performance across different configurations, ultimately contributing to its robust generalization capability.

### 3.4. Model Evaluation Criteria

A confusion matrix is needed to visualize a classification model’s effectiveness accurately. Each row represents the actual class, while each column represents the anticipated class, which is used to measure different evaluation parameters such as precision, recall, accuracy, and F1 score. Moreover, Receiver Operating Characteristic (ROC) curves for each class were also generated to evaluate the models’ performances. Attention heat maps were generated to identify which features the network paid more attention to while performing the classification task. Gradient-weighted Class Activation Mapping (GradCAM) also visualized class activation maps.

## 4. Experimental Results and Evaluation

The proposed MVSA-CNN model was evaluated based on its predictive performance, attention heatmap, and comparison with other state-of-the-art approaches. Furthermore, the model’s performance using various modifications in the network was also performed.

### 4.1. Visualization of Model Interpretability

**Figure 6:**
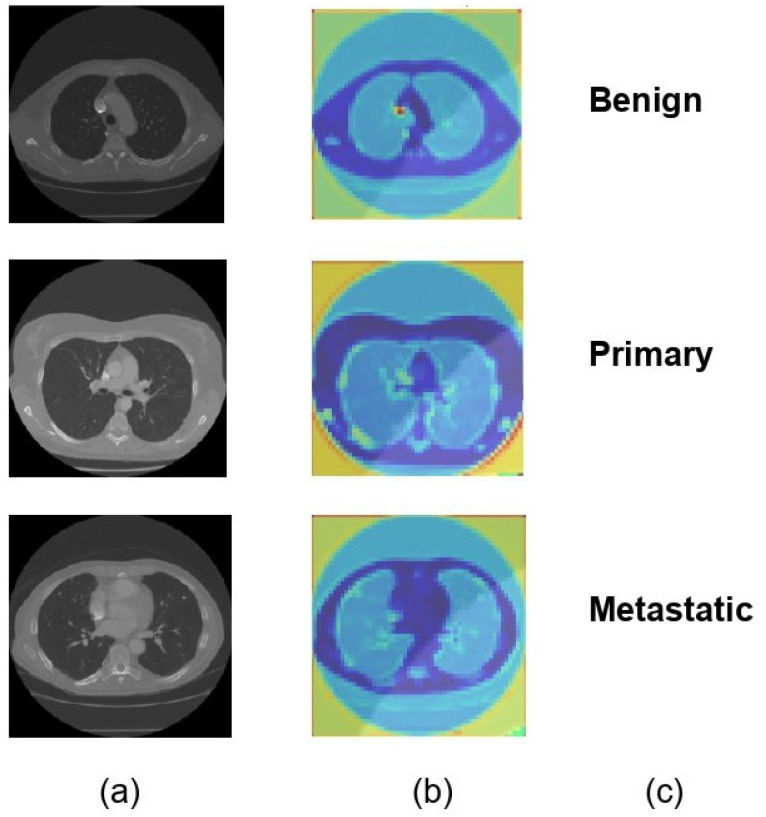
Visual representation of model classification with soft attention heatmaps for different types of lung nodules. (**a**) Original CT scans of lung nodules: benign, primary malignant, and metastatic. (**b**) SA heatmaps showing model focus areas for classification. (**c**) Final model predictions, confirming accurate identification of each nodule type.

For each of the three distinct classifications of lung nodules, benign, primary malignant, and metastatic malignant, Figure 6 provides a visual comparison of the input CT scan pictures, the matching Soft Attention (SA) heatmaps, and the final classification labels. The original CT scans, or raw data supplied into the model, are shown in column (a). Column (b) displays the SA heatmaps, which show the regions inside the images that the model prioritizes during classification. These intermediate findings, which precisely emphasize the important locations corresponding to each nodule type, demonstrate the interpretability of the suggested model. The final classification labels that the model assigned are shown in Column (c), demonstrating that it was able to accurately identify each type of nodule based on the features it had learned. The model’s predictive accuracy was improved overall by this visualization, which not only shows how well it can categorize lung nodules but also offers insights into the model’s decision-making process. The attention maps and classification results exhibit a clear alignment, indicating that the model concentrated on clinically significant aspects. This might make the model a useful tool for assisting with diagnostic decisions in medical imaging.

### 4.2. Performance of the Model

A confusion matrix, classification report, class activation heatmap, and soft attention map were adopted to visualize the model’s performance. The results presented in this section are based on 10-fold cross-validation.

### 4.3. Results of the Model’s Testing Using 10-Fold Cross-Validation

A 10-fold cross-validation approach was adopted to assess the model’s performance. The average of each performance metric was computed after the 10-fold cross-validation to arrive at a fair evaluation of the model’s performance. Table 4 demonstrates how efficiently the suggested models performed regarding average classification accuracy, specificity, and sensitivity. According to Table 4, the model attained the greatest values of 97.10%, 96.31%, and 97.45% for accuracy, sensitivity, and specificity, respectively.

**Figure 7:**
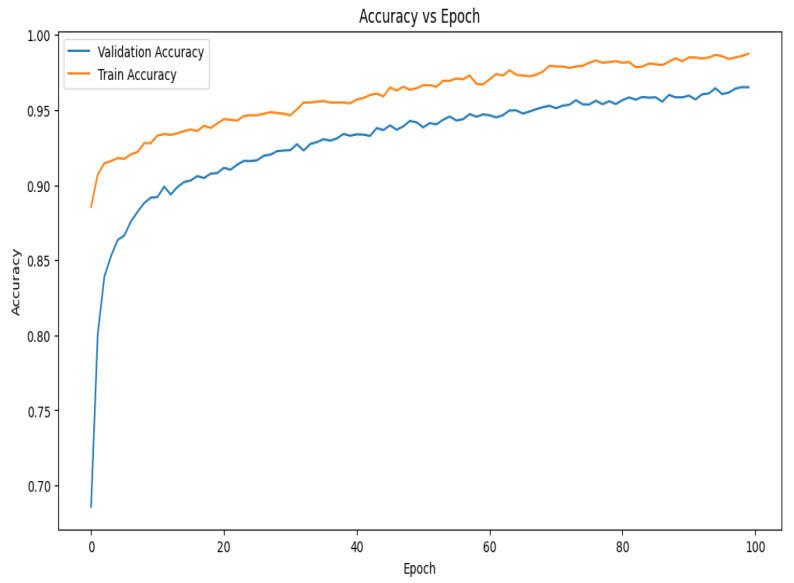
Accuracy vs. epoch graph of the proposed model for 10-fold cross-validation.

The accuracy vs. epoch graph shown in Figure 7 illustrates the model’s performance over 100 epochs. Although the training accuracy starts at a higher position and improves faster than validation accuracy, both metrics grow swiftly in the beginning. In the early phases of training, this shows that the model is effectively learning patterns in the training data. Demonstrating the model’s capacity to generalize effectively to new data, the validation accuracy also experiences a dramatic increase at first before stabilizing around the 30th epoch. The validation accuracy develops more smoothly on the present curve than it did on earlier trends, suggesting that the model is consistently generalizing over epochs. The gap between the training and validation accuracy remains relatively small, implying that the model is experiencing minimal overfitting and is able to maintain high accuracy on both datasets throughout the training process.

**Figure 8:**
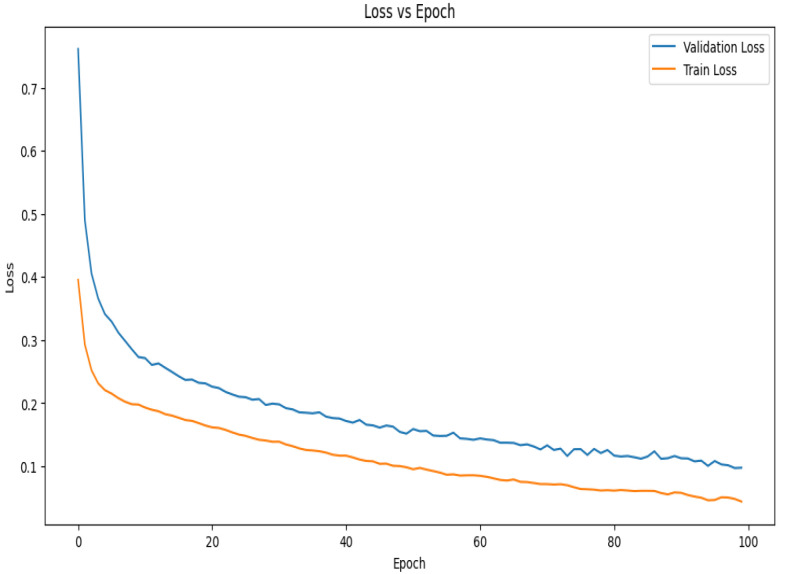
Loss vs. epoch graph of the proposed model for 10-fold cross-validation.

The loss vs. epoch curve for the suggested model during 10-fold cross-validation is shown in Figure 8. The model is learning rapidly and minimizing mistakes early in the training phase, as seen by the initial sharp decreases in both the training and validation losses. Up until about the 30th epoch, the validation loss declines steadily before plateauing and seeing very slight variations. This stability shows that the model is efficiently minimizing error and has successfully learned the distribution of the data. Given that the model has been exposed to training data, it is predicted that the training loss would exhibit a similar pattern to the validation loss but stabilize at a lower value. The constantly low validation loss suggests strong generalization power, even in the face of little differences in both curves. The curves together point to a well-tuned model with little overfitting that captures data patterns effectively and continues to perform well on unobserved data.

#### 4.3.1. Confusion Matrix and Classification Report

**Figure 9:**
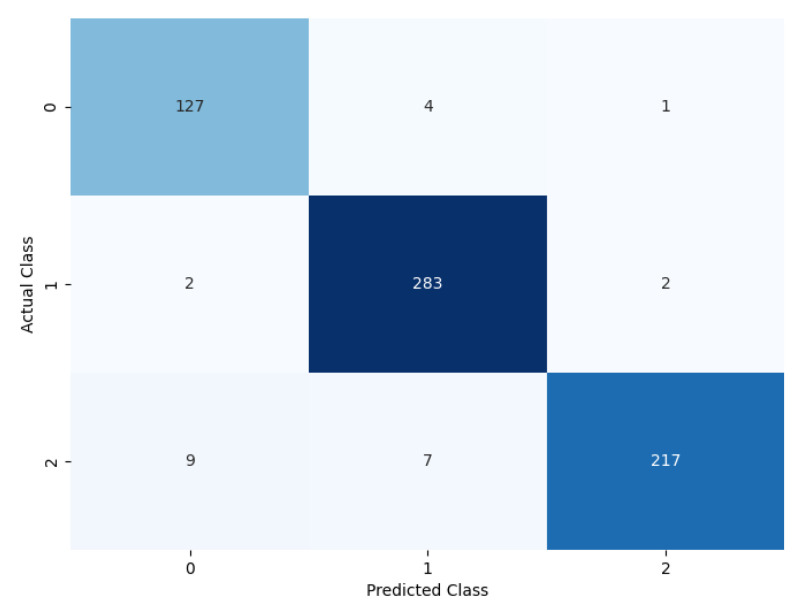
Confusion matrix of the proposed model.

The performance of the proposed model can be evaluated based on the confusion matrix in Figure 9, where for each of the classes, the prediction classification result can be observed. Out of 116 benign nodules, 111 were predicted as benign, 4 were predicted as primary, and 1 was predicted as metastatic.

Furthermore, out of 287 primary nodules, 2 were classified as benign, 283 were classified as primary, and 2 were classified as metastatic. Moreover, out of 236 metastatic nodules, 9 were classified as benign, 7 were classified as metastatic, and 220 were classified as metastatic.

A classification report based on the performance evaluation metrics for the classification model is given in Table 5. It measures different evolution parameters, such as precision, recall, accuracy, F1 score, and support. Each of the parameters achieves a good outcome for each of the classes. It shows the overall evolution parameter rate for the multi-class classification.

**Figure 10:**
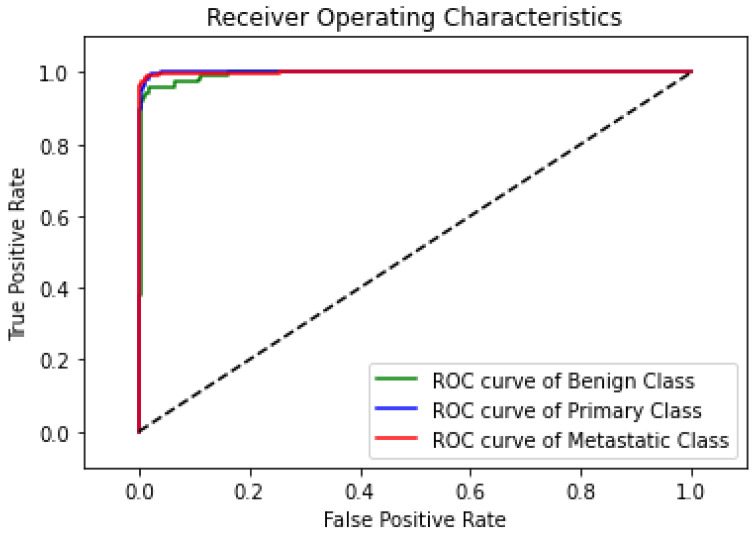
ROC curve of the proposed model.

The ROC curve, as shown in Figure 10, demonstrates the highly predictive ability of the proposed model in classifying lung nodules into benign, primary, and metastatic stages. The classifier’s decision threshold was changed in increments of 0.01, from 0 to 1, while the other hyperparameters remained fixed to produce the curves. The primary class’s ROC curve has a nearly flawless trajectory, whereas the benign class’s ROC curve displays a minor disparity because five nodules were incorrectly classified as primary or metastatic. In spite of this, the statistical data demonstrate the remarkable predictive power of the suggested model, as it distinguishes between the three nodule phases almost perfectly.

#### 4.3.2. Comparison between Class Activation Map and Soft Attention Map Visualization

**Figure 11:**
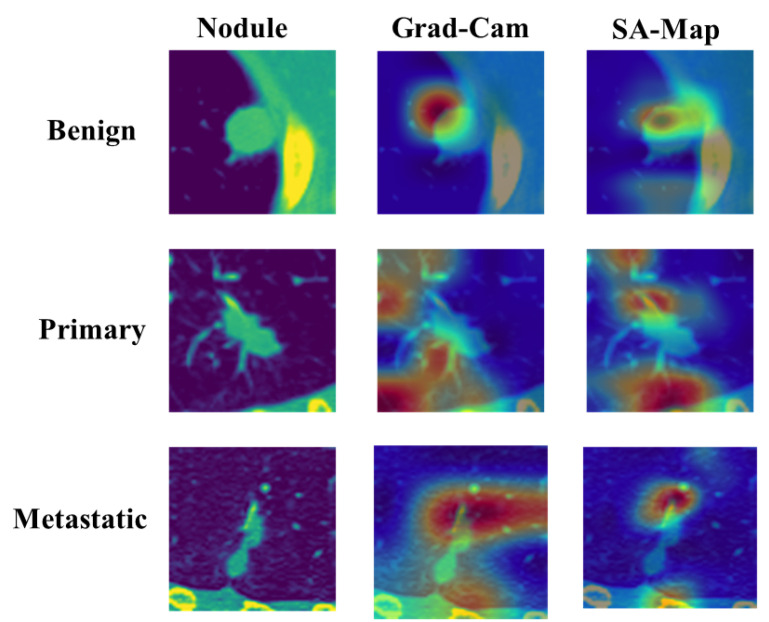
Comparison of GradCAM and soft attention heatmap.

Figure 11 shows the comparison between GradCAM class activation visualization and the soft attention heatmap. The original input image is shown in the first column, and to improve viewing and emphasize areas of interest, a color map was added. The heatmap of matching class activation created by GradCAM is shown in the second column, and the heatmap of soft attention is shown in the third. The elements the model concentrated on, shown in order to properly identify the class, are displayed in the attention heatmap. In comparison to the GradCAM visualization, it is evident that the soft attention heatmap is more focused and correctly identifies significant locations. In primary and metastatic nodules, when the presence of speculation and calcification is suggestive of malignancy, this concentrated attention is most noticeable. These properties are well recognized by the soft attention mechanism, confirming its efficacy in enhancing model interpretability and classification performance.

### 4.4. Performance Analysis of the Model through Network Modification

For a robust network architecture, it is essential to try different modifications in its structure to find the optimal one in terms of predictive performance. Experiments with some modifications, such as introducing soft attention at different depths of the network, with or without augmented data, removing the soft attention result, etc., were performed to understand their impact on the model.

#### 4.4.1. Performance of the Model without Soft Attention Layer

**Figure 12:**
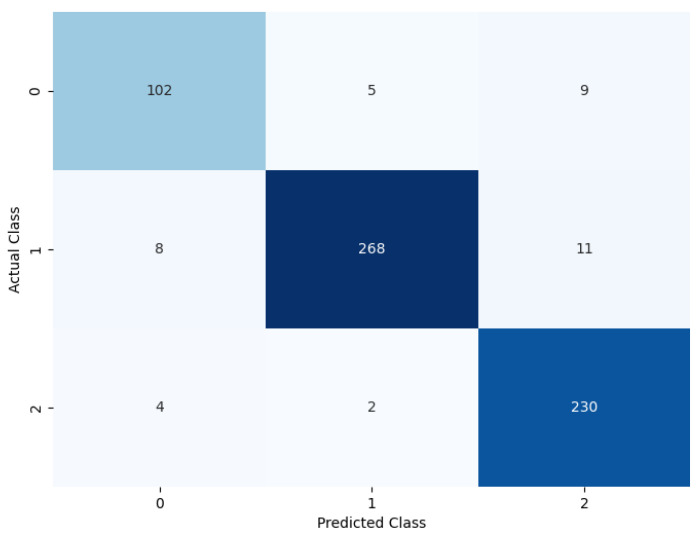
Confusion matrix of the model without soft attention.

The model was evaluated by removing the soft attention layer to understand its effect. The soft attention layer pays attention to the feature that the network uses to distinguish between nodule classes. In this experimental setup, the soft attention block was excluded to understand its effect. Table 6 shows the performance comparison between the model without the soft attention block and the model with the soft attention block. The confusion matrix is given in Figure 12.

Table 6 displays the model’s performance without the soft attention layer.

#### 4.4.2. Soft Attention at Different Depths of the Network

The number of feature sizes is used as input to the soft attention layer, which immediately influences the model’s performance. Table 7 illustrates the model’s performance when the soft attention layer input feature size is 16 × 16, 8 × 8, and 4 × 4.

#### 4.4.3. Performance of the Model without Using Custom Class Weights

**Figure 13:**
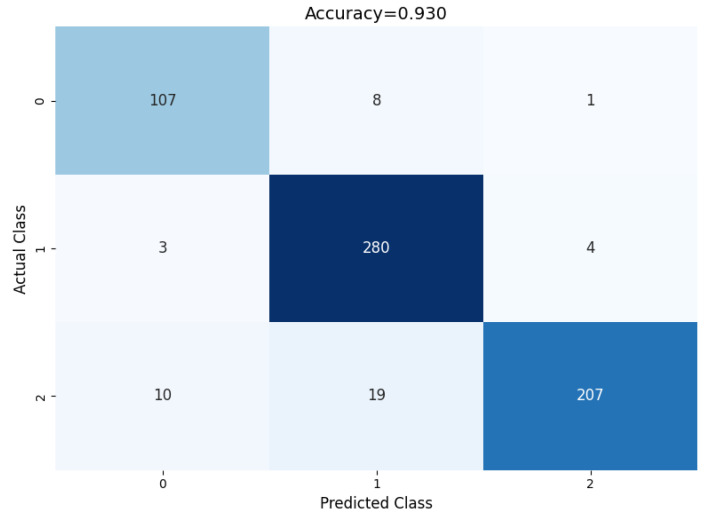
Performance of the model without using custom weights.

The proposed model shows a significant difference in behavior if all class weights are set to equal. The confusion matrix illustrated in Figure 13 shows the model’s performance if custom class weights are not set.

If all class weights are set to equal, the model tends to misclassify many of the benign and metastatic nodules. To tackle this problem, after empirical evaluation, the class weights of benign, primary, and metastatic were set to 2.5, 1.00, and 1.14, respectively, based on our empirical results. This change in class weight resulted in major performance improvement. The confusion matrix shows a summary of prediction results on the model’s performance without using custom class weights. The number of right and wrong predictions is summarized with count values and divided by class. The comparison of the model performance with and without custom class weights is shown in Table 8, using a confusion matrix to measure different evaluation parameters.

#### 4.4.4. Performance of the Model before and after Data Augmentation

The quantity of training images considerably influences the suggested model’s performance. The model overfits significantly without data augmentation of the training data. Table 9 shows the performance comparison of the model with and without data augmentation.

### 4.5. Comparison of the Model with Other State-of-the-Art Models

We evaluate the proposed model with the nodule classification methods introduced in recent years to observe comparative predictive performances. Table 10 provides the results of comparing classical classification approaches from different CNN-based detection methods. In terms of precision, recall, and specificity, Table 10 depicts the comparison between the proposed model MVSA-CNN to state-of-the-art classical lung nodule classification techniques in the LIDC-IDRI database.

Table 10 demonstrates that in terms of classification accuracy, sensitivity, and specificity, the suggested MVSA-CNN model outperforms the majority of conventional lung nodule classification techniques. The model outperformed numerous other models, with an accuracy of 97.10%, sensitivity of 96.31%, and specificity of 97.45%. In contrast, LungNet [29] produced comparable results in terms of accuracy and sensitivity, with 96.81% and 97.02%, respectively. However, the specificity of the suggested model was improved by 0.8%. This shows that our methodology is more successful in lowering false positives, which enhances the accuracy of identifying benign and malignant nodules. The suggested model shows a significant increase, especially in specificity, when compared to other high-performing models like FPSOCNN [24], which has an accuracy of 94.97%, and the model of Xie et al. [19], which has an accuracy of 93.40%. These findings demonstrate how well a multi-view method combined with a soft attention mechanism works to improve the model’s capacity to discriminate between different kinds of lung nodules. The suggested model combines extra contextual information from various perspectives and concentrates on essential aspects, resulting in more accurate and dependable nodule categorization, as seen by its improvements over conventional approaches and other sophisticated models.

## 5. Conclusions

In this study, we proposed a novel MVSA-CNN for the classification of lung nodules into benign, primary malignant, and metastatic malignant categories using CT images. The incorporation of a soft attention mechanism enabled the model to prioritize features that significantly contribute to malignancy while reducing the impact of irrelevant information, thus enhancing interpretability and classification accuracy. Our model achieved superior performance, with an accuracy of 97.10%, sensitivity of 96.31%, and specificity of 97.45%, outperforming existing state-of-the-art models. Even with these encouraging outcomes, there are a few restrictions to take into account. Firstly, the LIDC-IDRI dataset was the only one used to train and verify the model, so it might not accurately represent the variety of different populations or imaging modalities. Secondly, the model’s performance may be impacted by the dataset’s underrepresentation of various age groups, genders, and races. Next, the model’s application to other forms of lung cancer, such Small Cell Lung Cancer (SCLC), is limited since it focuses exclusively on NSCLC. Finally, the complex architecture with soft attention mechanisms requires significant computational resources, which could be challenging for use in resource-limited settings. To address these limitations, future work will focus on several key areas. Firstly, we will evaluate the model on a wider range of datasets, including those with varied patient demographics and imaging modalities, in order to reinforce the evaluation of the model and examine its resilience across different populations. Its predicted accuracy will be further improved by using clinical data and broadening the scope to include additional forms of lung cancer, such as SCLC. We also aim to simplify the model architecture, reducing its computational complexity to facilitate implementation in resource-limited settings. The model’s reliability in practical applications will be confirmed by extensive external validation utilizing separate datasets. In summary, the proposed MVSA-CNN model presents a significant advancement in the field of lung nodule classification, particularly in its ability to leverage multi-view inputs and soft attention mechanisms for improved diagnostic accuracy. By addressing the aforementioned limitations and expanding the model’s applicability, it has the potential to become a valuable tool for assisting radiologists in the early detection and accurate classification of lung cancer, ultimately improving patient outcomes. 

## Figures and Tables

**Table 1:** Summary of related works on lung nodule classification and their limitations.

Method	Author	Contribution	Limitations
ML	Setio et al. (2016) [11]	Proposed a ConvNets stream fed with six different views of lesion candidates for nodule classification. Utilized 3D data of nodules for training.	Increased network complexity; only features previously not taken from patches were incorporated, which could improve performance.
DL	Zhang et al. (2019) [9]	Developed CAD systems aiding radiologists in nodule identification. Provided a comparison viewpoint during the examination.	Less accurate performance in detecting tiny nodules.
DL	Sohan and Abu Yusuf (2020) [28]	Offered MED-GAN architecture for generating high-quality 3D bone shapes from 2D images using GAN and convolution networks.	Small dataset size and noisy images led to hazy output images.
DL	Masood et al. (2018) [35]	Developed a decision support system using DFCNet classifier on FCNN for lung cancer classification.	Suggested model is effective in labs but needs validation against existing CNN algorithms for real-world use.
DL	Nasrullah et al. (2019) [22]	Used faster R-CNN with features from CMixNet and U-Net for lung nodule detection.	High false positives and misdiagnoses due to different forms of errors.
DL	Tran et al. (2019) [15]	Developed 2D CNN (LdcNet) for lung nodule candidate identification in CTscans.	Needs to utilize the 3D character of lung lesions for improved performance.
DL	Silva et al. (2017) [8]	Developed CNN-based CADx system for lung nodule classification, removing the need for feature extraction and selection processes.	Need for more testing with other databases for better robustness and generalization.
DL	Polat and Danaei Mehr (2019) [20]	Presented 3D-GoogleNet and 3D-AlexNet for lung cancer detection inCTscans.	Handcrafted features are complicated and require significant time for extraction.

**Table 2:** Hounsfield units of different substances.

Substance	HU
Air	−1000
Water	0
Fat	−100 to +5
Lung	−700 to +600
Blood	+13 to +75
Muscle	+10 to +40
Soft tissue, contrast	+100 to +300
Cancellous bone	+300 to +400
Cortical bone	+1800 to +1900

**Table 3:** Training parameters.

Parameter	Value
Dataset	LIDC-IDRI
Train-test split ratio	80:20
Train–validation split ratio	80:20
Optimizer	Adam
Initial learning rate	0.0003
Epsilon	1 × $10^{-8}$
Class weights	Benign: 2.5
	Primary: 1
	Metastatic: 1.14
Loss function	Categorical cross-entropy
Mini batch size	64
Initial epochs	150
Early stopping	25 epochs
Min delta for early stopping	0.001
Number of classes	Benign
	Primary
	Metastatic

**Table 4:** Results of the model’s testing using 10-fold cross-validation.

Fold	Accuracy (%)	Sensitivity (%)	Specificity (%)
Fold 1	93.00	88.04	95.85
Fold 2	97.00	97.44	96.79
Fold 3	98.00	97.44	98.26
Fold 4	98.50	97.11	99.00
Fold 5	97.00	99.00	96.28
Fold 6	98.00	99.00	97.56
Fold 7	98.50	97.11	99.00
Fold 8	97.50	97.46	97.52
Fold 9	96.50	97.63	95.85
Fold 10	97.00	92.88	98.34
Average	97.10	96.31	97.45

**Table 5:** Classification report.

Class	Precision	Recall	F1 Score	Support
Benign	0.955	0.934	0.944	136
Primary	0.986	0.979	0.982	289
Metastatic	0.986	0.995	0.991	218

**Table 6:** Performance of the model without soft attention layer.

Model	Accuracy	Sensitivity	Specificity	FPR	FNR
Without SA	93.91%	92.92%	96.91%	3.09%	7.08%
With SA	97.10%	96.31%	97.45%	2.07%	3.92%

**Table 7:** Performance of the model using soft attention at different network depths.

SA Feature Size	Accuracy	Sensitivity	Specificity	FPR	FNR
SA-16 × 16	92.00%	91.92%	92.47%	7.53%	8.08%
SA-8 × 8	97.10%	96.31%	97.45%	2.07%	3.92%
SA-4 × 4	96.05%	95.71%	96.59%	3.41%	4.29%

**Table 8:** Comparison of model performance with and without custom class weights.

Model	Accuracy	Sensitivity	Specificity	FPR	FNR
Without custom class weights	93.04%	91.11%	93.32%	6.67%	8.89%
With default class weight	97.10%	96.31%	97.45%	2.07%	3.92%

**Table 9:** Performance of the model without data augmentation.

Model	Accuracy	Sensitivity	Specificity	FPR	FNR
Without augmentation	80.53%	78.01%	81.47%	18.53%	21.99%
With augmentation	97.10%	96.31%	97.45%	2.07%	3.92%

**Table 10:** Comparison with other state-of-the-art models.

Model	Accuracy	Sensitivity	Specificity
MV-KBC [30]	91.60	86.52	94.00
MK-SSAC [23]	92.53	84.94	96.28
DFCNet [35]	86.02	80.91	83.22
J.Lyu et al. [13]	92.19	92.10	91.50
CMixNet [22]	88.79	93.97	89.83
Xie et al. [19]	93.40	91.43	94.09
LungNet [29]	96.81	97.02	96.65
MC-CNN [14]	87.14	77.00	93.00
FPSOCNN [24]	94.97	96.88	95.89
Rehman et al. [31]	93	86	95.4
Mohamed et al. [41]	93.21	90.71	100
The proposed model	97.10	96.31	97.45

## Data Availability

All the data used for this research were collected from publicly available repositories.
